# Surface-Modified Industrial Acrylonitrile Butadiene Styrene 3D Scaffold Fabrication by Gold Nanoparticle for Drug Screening

**DOI:** 10.3390/nano10030529

**Published:** 2020-03-15

**Authors:** Kaudjhis Patrick Ulrich N’deh, Gyeong-Ji Kim, Kang-Hyun Chung, Jae-Soo Shin, Kwang-Sup Lee, Jeong-Woo Choi, Kwon-Jai Lee, Jeung Hee An

**Affiliations:** 1 Department of Food Science and Technology, Seoul National University of Science & Technology, Seoul 01811, Korea; kaudjhispatrick@gmail.com (K.P.U.N.);; 2 Department of Food Science and Nutrition, KC University, Seoul 07661, Korea; kgj8495@hanmail.net; 3 Department of Biomedical Engineering, Sogang University, Seoul 04107, Korea; 4 Department of Advanced Materials Engineering, Daejeon University, Daejon 34520, Korea; jsshin@dju.ac.kr; 5 Department of Advanced Materials, Hannam University, Daejeon 34520, Korea; kslee@hnu.kr; 6 Department of Chemical and Biomolecular Engineering, Sogang University, Seoul 04107, Korea; jwchoi@sogang.ac.kr; 7 Department of H-LAC, Daejeon University, Daejon 34520, Korea

**Keywords:** 3D cell culture, industrial ABS scaffolds, gold nanoparticles, cancer cells, skin cells

## Abstract

Biocompatibility is very important for cell growth using 3D printers, but biocompatibility materials are very expensive. In this study, we investigated the possibility of cell culture by the surface modification of relatively low-cost industrial materials and an efficient three-dimensional (3D) scaffold made with an industrial ABS filament for cell proliferation, spheroid formation, and drug screening applications. We evaluated the adequate structure among two-layer square shape 3D scaffolds printed by fused deposition modeling with variable infill densities (10–50%). Based on the effects of these scaffolds on cell proliferation and spheroid formation, we conducted experiments using the industrial ABS 3D scaffold (IA3D) with 40% of infill density, which presented an external dimension of (XYZ) 7650 µm × 7647 µm × 210 µm, 29.8% porosity, and 225 homogenous micropores (251.6 µm × 245.9 µm × 210 µm). In the IA3D, spheroids of cancer HepG2 cells and keratinocytes HaCaT cells appeared after 2 and 3 days of culture, respectively, whereas no spheroids were formed in 2D culture. A gold nanoparticle-coated industrial ABS 3D scaffold (GIA3D) exhibited enhanced biocompatible properties including increased spheroid formation by HepG2 cells compared to IA3D (1.3-fold) and 2D (38-fold) cultures. Furthermore, the cancer cells exhibited increased resistance to drug treatments in GIA3D, with cell viabilities of 122.9% in industrial GIA3D, 40.2% in IA3D, and 55.2% in 2D cultures when treated with 100 µM of mitoxantrone. Our results show that the newly engineered IA3D is an innovative 3D scaffold with upgraded properties for cell proliferation, spheroid formation, and drug-screening applications.

## 1. Introduction

Scaffold-based three-dimensional (3D) cell cultures are important technologies with numerous applications in drug development, tissue engineering, and regenerative medicine [1,2]. These innovative biomaterials have brought advances to and broaden the possibilities of cellular and molecular biology. The microenvironment offered by 3D scaffolds promotes cells adhesion to the extracellular matrix, cell-to-cell interaction, cell growth in 3D shapes, and cell differentiation [3,4]. The main advantage of 3D scaffolds is to reproduce reliable in vitro models able to mimic the in vivo conditions for tissue engineering and new drug development applications [1]. Nowadays, the development of modern drugs requires the implementation and validation of a wide range of preclinical tests and protocols that are costly and time-consuming [5]. A usual process of preclinical drug testing requires both cell and animal experiments. Unfortunately, the traditional in vitro two-dimensional (2D) cell cultures poorly recapitulate the in vivo environment and have many limitations, including an altered cell morphology, polarity, phenotypes, and division mode in cells, as well as a disturbance of cell-to-cell and cell-to-extracellular environment interactions [6]. Since these models are better suited for understanding the overall effects of an experiment on a living subject [7], these models often make it difficult to understand the drug-specific mode of action [8]. Three-dimensional models such as 3D scaffolds better mimic the in vivo conditions for cell studies, tissue organization, and drug screening applications, by comparison to conventional 2D models. Therefore, they can be considered as potent alternatives to animal testing. Several materials are used in 3D scaffolds printing [9,10,11,12,13,14].

The biocompatible materials commonly used in 3D scaffold printing are polylactic acid (PLA), polyglycolic acid (PGA), polycaprolactone (PCL), and polyethylene glycol (PEG) [9]. Alternatively, the most commonly used materials by 3D printers are PLA and acrylonitrile butadiene styrene (ABS) [9]. For biological applications, the thermoplastic bio-ABS material type has been used to create ear-shaped molds for human skin cell culture and to create tympanic membranes using bovine articular chondrocytes [10,11]. In addition, the biocompatible material ABS-M30i has been used to create a new generation of biocompatible and biomimetic implants, which can be used for the replacement of not only bone segments but also entire bones [12]. However, apart from ABS-M30i, which is suitable for biotechnological applications, industrial ABS is known to be non-biodegradable and non-biocompatible [9]. Various polymers, such as PLA, PCL, PEG, and ABS have been used in 3D scaffold fabrication for cell culture and tissue engineering applications. However, industrial ABS has not been evaluated with respect to its biocompatibility or toxicity.

We printed a 3D scaffold using industrial ABS material and coated that structure with gold nanoparticles (Au-NPs) to increase the cellular safety of the industrial ABS material. According to a recent study, the development of 3D nanocomposite materials with incorporated nanoparticles has attracted interest, as nano-sized particles offer the potential to enhance various properties of the 3D-printed parts [13,14]. Au-NPs are widely used in biological research, particularly in cell-based assays, due to their biocompatibility, unique surface plasmon, optical and catalytic properties, high chemical stability, suitable surface functionality, ease of functionalization, and increased mechanical properties [15,16]. Au-NPs have been incorporated into macroporous scaffolds to increase matrix conductivity and to promote cell adhesion, growth, differentiation, maturation, and morphogenesis [15,16,17,18,19].

In this study, we investigated a low-cost industrial ABS 3D scaffold (IA3D) printed by fused deposition modeling for cell proliferation, spheroid formation, and drug screening. To improve the biocompatible properties of the industrial ABS material, Au-NPs were coated on the IA3D. Our result is the first report of a gold nanoparticle-coated industrial ABS scaffold (GIA3D), which can be easily employed to stimulate cell viability and proliferation *in vitro*, as well as to mimic the in vivo conditions for drug testing.

## 2. Materials and Methods 

### 2.1. Fabrication and Characterization of IA3D

**Figure 1:**
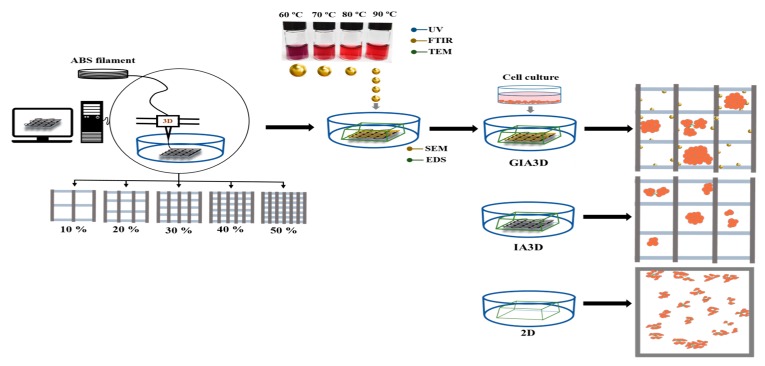
Schematic diagram of the experimental method. The 3D scaffold patterns were designed using a computer with NewCreatork program (version 1.57.41) for 3D printers. Industrial acrylonitrile butadiene styrene (ABS) white filament (1.75 mm) was inserted in the extruder feed throat of a 3D bioprinter and heated at 250 °C in the chamber. The scaffold was printed layer-by-layer via the fused deposition modeling technique into a 60-mm format Petri dish. A 3D culture microenvironment was created by fixing a miniature plastic chamber (XYZ approximately 1 cm × 1 cm × 1 cm) around the scaffold, in which gold nanoparticles (Au-NPs) were chemically synthesized and coated on the scaffold’s structure by deposition. The synthesis of Au-NPs was realized by the reduction of Au^3+^ ions from gold (III) chloride with the citrate from trisodium citrate at high temperature (60 °C to 90 °C) for 60 min of reaction. Finally, the cancer model HepG2 and keratinocyte HaCaT cells were cultured in 3D (with/without Au-NPs) and 2D culture systems (same size miniature plastic chamber was fixed on the flat surface of petri dish).

The fabrication of the IA3D is schematically presented in Figure 1. Briefly, the two-layer 3D scaffold with a square shape pattern (XYZ) (8 mm × 8 mm × 0.2 mm) was produced using the computer-aided design (CAD) software NewCreatork (version 1.57.41) and printed in a 60-mm format petri dish using a fused deposition modeling (FDM) technique with a 3D bioprinter (Rokit In vivo, Seoul, Korea). The optimal position of the petri dish was selected on a XYZ piezoelectric stage under the 200 μm extruder nozzle. Subsequently, the IA3D white filament (1.75 mm) acquired from 3D KNT (Seoul, Korea) was inserted into the extruder feed throat and printed by extrusion at 250 °C in the petri dish. The main printing parameter was the infill density. In the FDM technique, the infill density determines the amount of material that is filled into an object [20]. Modifications in the infill density parameter alter 3D printed structures, leading to printed objects with various porosities [9]. IA3D with 10%, 20%, 30%, 40%, and 50% infill densities were printed within 2 min with printing speed set to 5 mm/s and filament input flow fixed to 200%.

The morphology and microstructure of the scaffolds was visualized under an optical microscope (Nikon Eclipse TS100led Trinocular) equipped with a digital camera and with scanning electron microscope (SEM) (ZEISS GEMINI2, Oberkochen, Germany). The approximate average dimensions of rectangular pores (XY) for each scaffold group were calculated from the images made by the optical microscope via ImageJ software (NIH 1.51a), (*n* ≥ 5) [21]. The scaffold porosity (in volume %) was measured using the following equation [22]:

(1) $$\mathrm{Porosity}\;(\%)=\frac{V-\left(\frac{M}{\rho }\right)}{V}\times 100\%,$$

where *V* is the volume of the scaffold calculated using its outer dimension, *M* is the mass of the porous scaffold, and *ρ* is the density of IA3D (1.04 g/cm^3^). Five scaffolds per infill density type were dried overnight at 80 °C and weighed (*M*). Then, the porosity of scaffold was calculated using Equation (1).

### 2.2. Au-NPs Synthesis, Characterization, and Surface Modification of Scaffold

To enhance the biocompatibility of IA3D, Au-NPs were coated onto IA3D by deposition. First, a miniature plastic chamber system, approximately (XYZ) 1 cm x 1 cm x 1 cm (Lab-Tek chamber 177402, Thermo Fisher Scientific Inc, Rochester, NY, USA) was fixed around the printed scaffold with polydimethylsiloxane. Subsequently, Au-NPs were synthesized in the plastic chamber via a chemical method modified from Turkevich and Frens 1951, 1973 [23,24,25]. The reduction of auric ions (Au^3+^) from chloroauric acid trihydrate (HAuCl_4_.3H_2_O) (Kojima Chemicals, Japan) was initiated by the citrate from trisodium citrate dihydrate salt (C_6_H_5_Na_3_O_7_.2H_2_O) (Samchun Pure Chemicals, Pyeongtaek, Korea). Briefly, in the miniature plastic chamber, a 500 µL solution containing 0.25 mM of Au^3+^ and 1.5 mM of citrate (1:6) was prepared by diluting the corresponding salts in deionized water. Then, the mixture was stirred at 50 rpm on an orbital shaker for 5 min, and scaffolds were incubated in a preheated (approximately 35 °C) dry oven (SW-DO002, Gimhae, Seoul, Korea) at high temperature (60 °C, 70 °C, 80 °C, or 90 °C) for 60 min. Finally, scaffolds were cooled to 36 °C in the oven and then extracted. Synthesized Au-NPs coated the surface of IA3D by deposition, whereas the Au-NPs remaining in colloidal solution were stored in glass vials protected from light for further analysis, including UV-vis, Fourier-transform infrared spectroscopy (FTIR), and transmission electron microscopy (TEM).

The UV-vis transmittance spectra of Au-NPs in colloidal solutions synthesized at 60 °C, 70 °C, 80 °C, and 90 °C were recorded in the range between 380 and 780 nm using a Shimadzu UV mini-1240 spectrophotometer (Kyoto, Japan) in a 1-cm glass cuvette. The FTIR spectra of Au-NPs solution synthesized at 90 °C and trisodium citrate solution (88 g/L) were recorded without any sample preparation using a Cary 630 FTIR spectrometer in the spectral ranging from 650 to 4000 cm^–1^. Then, the IR spectra were collected using Agilent MicroLab PC software (Agilent Technologies, Inc., Santa Clara, CA, USA). The size of the particles was analyzed by TEM using a Hitachi HF-2000 (Tokyo, Japan) field emission TEM operating at 200 kV. From the TEM images, the sizes of particles in different samples were determined by counting at least 100 particles. The presence of Au-NPs on the surface of the IA3D was verified by energy-dispersive X-Ray spectroscopy (EDS) analyses with SEM.

### 2.3. Cell Seeding in IA3D and GIA3D

The 3D culture microenvironments were fabricated for both the IA3D and the GIA3D by fixing the miniature plastic chamber around the scaffold’s structure, as described previously in Section 2.2. Additionally, a 2D culture microenvironment was fabricated by fixing a plastic chamber of the same size on the flat surface of a Petri dish. HaCaT cells (Human keratinocyte cells) and HepG2 cells (human hepatoma cells) were obtained from the American Type Culture Collection (ATCC). Cells were cultured in Dulbecco’s modified Eagle’s medium (Invitrogen, Carlsbad, CA, USA) supplemented with 10% fetal bovine serum (Hyclone, Logan, UT, USA), 200 mM of glutamine, and 100 U/mL of penicillin in a humidified chamber with 5% CO_2_ at 37 °C.

### 2.4. Cell Morphology Cultured on Surface Coated with Au-NPs

The surface morphologies and metabolic status of HepG2 and HaCaT cells on a gold nanoparticles-coated 2D plate (G2D) and GIA3D were analyzed by SEM. The SEM image was used to compare the growth of HepG2 cells (seeded at 3 × 10^3^ cell/mL) and HaCaT cells (seeded at 5 × 10^4^ cell/mL) cultured on GIA3D and G2D at 48 h.

### 2.5. Cytotoxicity of IA3D and GIA3D

To select the IA3D with optimum infill density, a Live/Dead cell assay kit (BioVision) was used to qualitatively assess the viability and proliferation of the HaCaT cells (seeded at 5 × 10^4^ cell/mL) on IA3D printed with various infill densities (10–50%). This assay was also used to compare the growth of HepG2 (seeded at 3 × 10^3^ cell/mL) cultured on either a 2D plate, IA3D, or GIA3D at 3, 5, 7, 9, and 11 days. In addition, the total spheroid population of a 72 h HepG2 cell culture in IA3D versus GIA3D was determined using Live/Dead cell assay. In this assay, cell cultures were treated with Live-Dye, a cell-permeable green fluorescent dye, and propidium iodide, a non-permeable red fluorescent dye. Live cells were stained with only the cell-permeable fluorescent green dye, and dead cells were stained with both the cell-permeable Live-Dye and propidium iodide. The cells were observed immediately under a fluorescence microscope Nikon Eclipse TS100led Trinocular (Nikon corporation, Tokyo, Japan) using a band-pass filter (detect fluorescein and rhodamine).

In addition, the viability of HepG2 cells and HaCaT cells in the scaffolds was determined by a EZ-Cytox cell viability assay kit (DoGenBio Co. Ltd, Seoul, Korea), which is based on the cleavage of the tetrazolium salt to water-soluble formazan by succinate-tetrazolium reductase. HepG2 cells (1 × 10^3^, 2 × 10^3^, and 3 × 10^3^ cells/mL) and HaCaT cells (8 × 10^3^, 1 × 10^4^, and 5 × 10^4^ cells/mL) were cultured in 2D system, IA3D, and GIA3D for 6, 24, 48, and 72 h. Next, 10% of EZ-Cytox reagent was added to the media and incubated for 1 h at 37 °C. Subsequently, 100 µL of that media (in triplicate) was added per well in a 96-well plate, and absorbance was measured using micro-titer plate reader from 420–480 nm with a reference of 650 nm. Blanks were used for each concentration to ensure accurate results.

### 2.6. Measurement of Apoptosis

HaCaT cells were seeded in the 2D system, IA3D, and GIA3D at a density of 5 × 10^4^ cells/mL. Apoptotic cells were quantified using an Annexin V-FITC apoptosis detection kit (BD Biosciences, San Diego, CA, USA) according to the manufacturer’s instructions.

### 2.7. Immunofluorescence Staining

HaCaT cells (seeded at 5 × 10^4^ cell/mL) were plated on the GIA3D on day 3. Cells were stained using primary antibodies of F-actin and secondary antibodies labeled with Q-dot-conjugated fluorescein isothiocyanate (FITC, green; Invitrogen, Carlsbad, CA, USA). The cells were viewed and photographed using a confocal laser scanning microscope (Carl Zeiss MicroImaging, Oberkochen, Germany). The images were acquired using a confocal microscope with LSM imaging software (Carl, Zeiss, Jena, Germany).

### 2.8. Drug Screening in IA3D and GIA3D

We also measured the viability of cells intoxicated with asiatic acid 97% (Sigma-Aldrich, Saint Louis, MO, USA) and mitoxantrone (Reyon Pharmaceutical Co., Ltd., Seoul, Korea) to compare the drug-resistance capacity of HepG2 (3 × 10^3^ cell/mL) and HaCaT cells (5 × 10^4^ cell/mL) between the 2D system, IA3D, and GIA3D. After 72 h of culture, HepG2 cells were exposed to mitoxantrone (0, 1, 10, 50, and 100 µM) and asiatic acid (0, 10, 75, 150, and 200 µM) for 24 h, while the HaCaT were treated with mitoxantrone (0, 0.1, 0.3, 0.5, and 0.8 µM) and asiatic acid (0, 50, 100, 200, and 300 µM) for 24 h. Experiments were performed in triplicate, and the cytotoxic effects of the drugs were measured using the EZ-Cytox 3000 kit as described previously. Results from the cell viability experiments are expressed as the percentage of cell viability compared to that of the non-treated control.

### 2.9. Statistical Analysis

Statistical analysis was performed using SPSS 18.0 (SPSS Inc.). Averages and standard deviations were calculated and differences between groups were assessed using the analysis of variance method and Duncan’s multiple range test. Differential values were considered significant if *p* < 0.05.

## 3. Results and Discussion

### 3.1. Properties of IA3D

**Figure 2:**
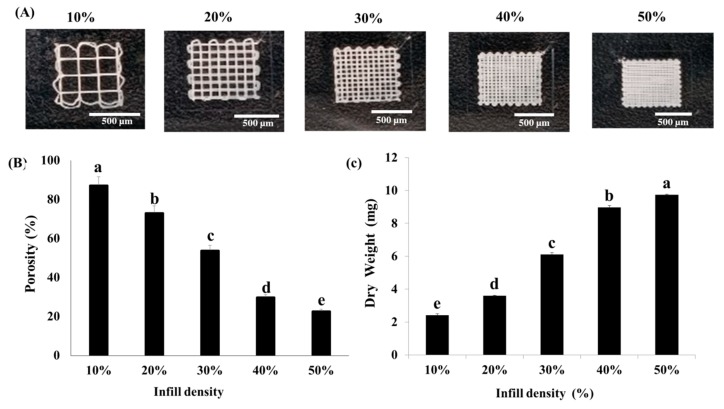
Characteristics of three-dimensional printed industrial acrylonitrile butadiene styrene scaffolds with different infill densities (10%–50%). (**A**) Photographs of industrial ABS scaffolds with infill densities ranging from 10% to 50%. Scale bar: 500 µm. (**B**) Measured porosity. (**C**) Measured dry weight. Results are expressed as mean ± SD. Significant differences (*p* < 0.05) are represented using different letters.

The infill density parameter ranges from 0% to 100%, where 0% of infill results in a completely hollow object and that of 100% results in a completely solid object [9]. By modifying the infill density parameter (10–50%), different 3D scaffolds were fabricated using IA3D (Figure 2A). The approximate height of 3D scaffolds was 209 ± 12 µm. The shapes of the resultant pores were rectangular and the average pore dimensions, which was also named the pore size (XY), were inversely correlated to the infill density. Indeed, pore size decreased from approximately 1890 µm × 1907 µm (10% of infill density), 740 µm × 752 µm (20% of infill density), 377 µm × 380 µm (30% of infill density), 248 µm × 250 µm (40% of infill density) to 161 µm × 168 µm (50% of infill density) (*n* ≥ 5), (Figure 2A). Further measurements of porosities showed a linear decrease from 83.3%, 73.1%, 53.8%, and 29.8% to 22.6% for the 3D scaffolds with infill densities of 10%, 20%, 30%, 40%, and 50%, respectively (*p* < 0.05) (Figure 2B). However, the dry weight of scaffolds linearly increased from 2.22 mg, 3.60 mg, 6.10 mg, and 8.97 mg, to 9.73 mg for the scaffolds with infill density of 10%, 20%, 30%, 40%, and 50%, respectively (*p* < 0.05) (Figure 2C). Studies have demonstrated that the porosity and pore size are some of the most significant characteristics of 3D printed scaffolds in tissue engineering [26,27,28,29,30,31,32]. In fact, scaffolds with adequate pore size and porosity provide a suitable microenvironment for sufficient cell–cell interaction and cell migration, proliferation, and differentiation [29]. It is also important to note that excessively small pores in scaffolds prevent cells from migrating in toward the center of the construct, consequently limiting the diffusion of nutrients and the removal of waste products. On the other hand, in larger pores (i.e., 325 µm as the mean pore size used for skin cell culture [27]), cell aggregations are reduced, and cell attachment is limited as a result of the decreased available specific area [28]. Moreover, Gregor et al. demonstrated that for bone tissue replacement, a porosity of 30% (and not 50%) is optimal for PLA scaffolds printed by the fused deposition modeling technique [33]. Indeed, the cultured osteosarcoma cell line MG-63 exhibited more successful proliferation and osteoconduction with only 30% porosity in comparison to the 50% porosity scaffold groups. Yang et al. investigated the optimal pore size (200, 350, or 500 μm) of bone tissue implants and found that the 350 μm scaffolds exhibited a better expression level of osteogenic genes [34]. In addition, the optimal pore size for ligament tissue ingrowth in braided ligament scaffolds in PLAGA 10:90 braids was found to be between 175 and 233 μm [35]. Therefore, to select the 3D scaffold with adequate porosity and pore size for the cell ingrowth preferentially in a spheroid shape, cell culture experiments were performed.

### 3.2. Optimization of the Infill Density and Cell Seeding Density in IA3D

**Figure 3:**
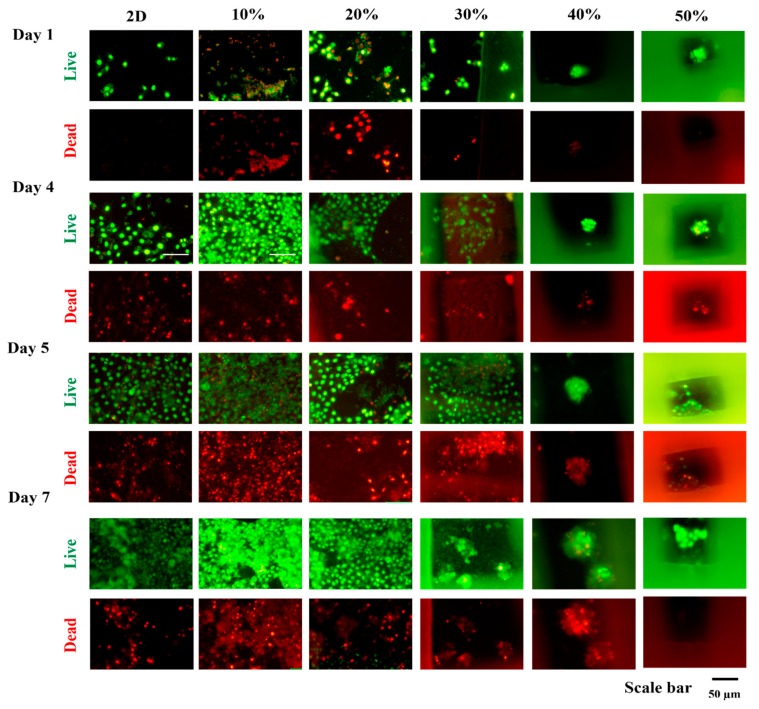
Live/Dead staining of keratinocytes human keratinocyte cells (HaCaT) cells cultured in three-dimensional printed industrial acrylonitrile butadiene styrene scaffold printed with different infill densities ranging from 10% to 50%. Cells were seeded at 5 × 10^4^ cell/mL; scale bar: 50 µm.

Live/Dead staining was performed to determine the scaffold with optimal infill density by seeding the human keratinocytes HaCaT cells at 5 × 10^4^ cells/mL in IA3D (10%, 20%, 30%, 40%, and 50% of infill density) (Figure 3). At day 1 of incubation, scaffolds printed with 10% and 20% infill densities exhibited higher toxicity to cells than the scaffolds with 30%, 40%, and 50% infill densities (Figure 3). At day 4 of incubation, dead cells were found in 2D culture systems, whereas HaCaT cells cultured in scaffolds with 30%, 40%, and 50% infill densities did not show dead cells. At day 5 of incubation, dead cells appeared in the scaffolds with 30% and 40% infill densities. Similar to the 2D culture, in scaffolds with 10% and 20% infill densities, cells were spreading on the internal flat surface of the pores, reducing the strength of cell–cell interactions. However, in scaffolds with 40% and 50% infill densities, tight structures of cells aggregates appeared from day 1 of culture, while the cell aggregates were formed later in the scaffolds with 30% infill density at day 7 of incubation. Overall, we found that the propensity to form 3D cell aggregates was highly correlated to the infill density. In this study, an IA3D with higher infill density (lower porosity) promoted cells’ aggregation, while the scaffolds with lower infill density (higher porosity) promoted cell spreading. These findings are in accordance with recent studies in which higher porosities were shown to promote cell spreading on the internal surface of bigger pores, whereas the lower porosities promoted 3D cell aggregation [30]. 3D scaffolds with an infill density of 50% performed better than those with an infill density of 40% in promoting cell aggregation, but it was difficult to observe the cells under microscope in IA3D with 50% infill because of the narrow pore size and high fluorescent signal density from stained cells. Recent studies in tissue engineering recommended scaffolds with pore sizes ranging from 200 to 300 μm for the growth of tissues such as fibrocartilaginous tissue [36]. It has been established that cell proliferation on scaffolds is highly correlated with the material, scaffold structure, and cell kinetics [37]. This study is the first report showing that scaffolds fabricated using industrial ABS filament are effective in promoting the viability and proliferation of cells. Therefore, we decided to conduct our experiments by using the IA3D fabricated with 40% infill density (porosity 29.8%, pore size: approximately 248 µm × 250 µm) (Figure 2).

### 3.3. Physical Properties of Au-NPs

To improve the biocompatible properties of the industrial ABS material, Au-NPs were synthesized at high temperature (60–90 °C) and coated on scaffolds (Figure 1). During synthesis, the color of the solution (gold [III] chloride and trisodium citrate) changed from colorless to purple and finally to ruby-red. These colors were thought to characterize the formation of gold nanoparticles in solution [38]. Further optical tests such as UV-vis and TEM assessments were performed to confirm these findings.

**Figure 4:**
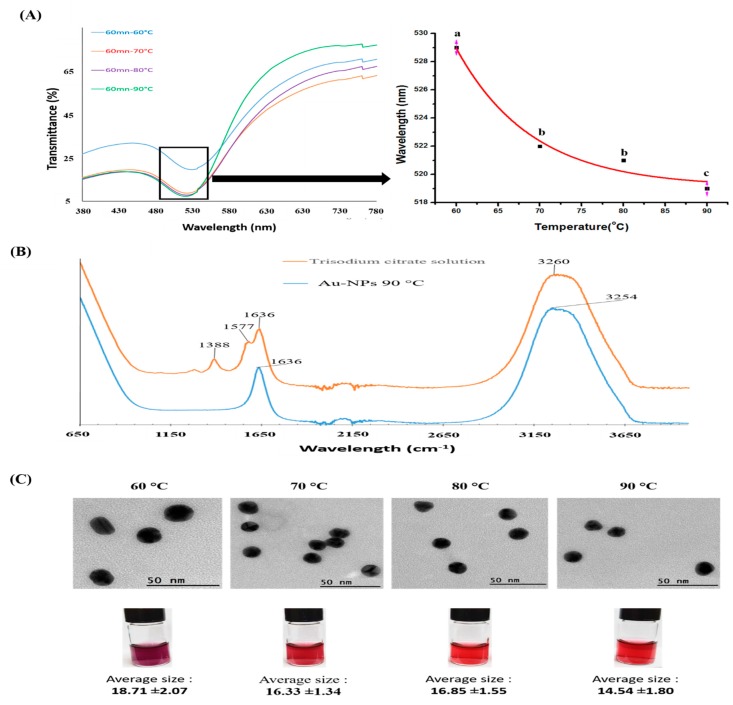
Optical characterization of gold nanoparticles (Au-NPs). (**A**) UV-vis transmittance spectra of Au-NPs colloidal solutions synthesized at high temperatures (60 to 90 °C) for 60 min. Results are expressed as mean ± SD, n = 3. Significant differences (*p* < 0.05) are represented using different letters (a, b, c). (**B**) The Fourier-transform infrared spectroscopy (FTIR) spectra of trisodium citrate solution (88 g/L) and Au-NPs colloidal solution synthesized at 90 °C for 60 min. (**C**) TEM micrographs of gold nanoparticles synthesized at 60 °C, 70 °C, 80 °C, and 90 °C corresponding to the respective photography of Au-NPs colloidal solutions. The mean sizes of all four samples were determined by counting at least 100 particles.

Figure 4A shows the UV-vis wavelength spectra of Au-NPs colloidal solutions recorded from 380 to 780 nm. The evolution of UV-vis peaks in Au-NPs synthesized at 60 °C, 70 °C, 80 °C, and 90 °C showed a decrease in the wavelength number associated with the increase in temperature. Indeed, the corresponding peaks of Au-NPs peaks synthesized at 60 °C, 70 °C, 80 °C, and 90 °C were recorded as 529, 522, 521, and 519 nm, respectively. A similar conclusion was reached by Minh Tran et al. when synthesizing Au-NPs by the Turkevich approach [23,24,25]. They showed that temperature strongly influences the physical parameters of Au-NPs, including their corresponding UV-vis peaks and particle sizes [39]. In addition, according to the published literature, a lower wavelength value for the Au-NPs UV-vis peak is highly correlated with particles of a smaller diameter [40].

The FTIR spectra of trisodium citrate solution (88 g/L) and Au-NP suspension synthesized at 90 °C are presented in Figure 4B. The characteristic peaks of trisodium citrate solution at 1388 cm^−1^ and 1577 cm^−1^ correspond to the symmetric and anti-symmetric stretching of carboxylate (COO^-^). The peaks at 1636 cm^−1^ and 3254 cm^−1^ can be assigned to the O-H scissor bonds and O-H stretching bonds, respectively. These data confirm that citrate served as a capping agent in Au-NPs [41].

To further confirm the UV-vis results, TEM measurements were performed on Au-NPs samples synthesized from 60 to 90 °C (Figure 4C). TEM micrographs revealed the synthesis of spherical particles. The decrease in nanoparticle size was also inversely correlated with the temperature of the reaction. Average particle sizes were 18.71 ± 2.07 µm, 16.33 ± 1.34 µm, 16.85 ± 1.55 µm, and 14.54 ± 1.80 µm for nanoparticles synthesized at 60 °C, 70 °C, 80 °C, and 90 °C, respectively (Figure 4C). These findings indicate that Au-NPs could be effectively synthesized via the method modified from Turkevich and Frens (1951), (1973).

### 3.4. Characterization of Surface Modified GIA3D

**Figure 5:**
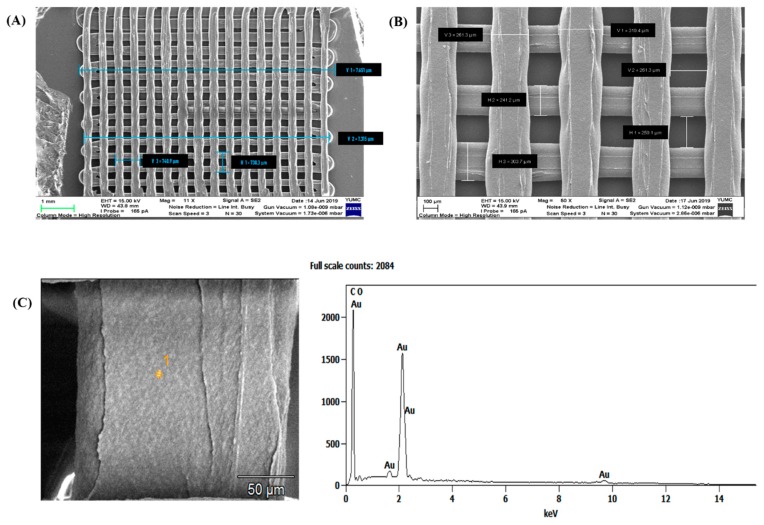
Optical characterization of the gold nanoparticles (Au-NPs)-coated industrial acrylonitrile butadiene styrene scaffold (GIA3D), printed with an infill density of 40%. (**A**) SEM images of the three-dimensional printed industrial acrylonitrile butadiene styrene scaffolds (IA3D) with 40% infill density showing the overall size. Scale bar 1 mm. (**B**) SEM images of the IA3D with 40% infill density showing pore size and fiber width. Scale bar: 100 µm. (**C**) Authentication of GIA3D with SEM, scale bar 50 µm. SEM, scanning electron microscopy; EDS, energy-dispersive X-ray spectroscopy.

Figure 5A,B show the SEM micrographs of the optimal scaffold (40% infill density model). The overall size of the printed scaffold was 7650 ± 37.2 µm × 7647 ± 41.9 µm × 210 ± 9 µm, which is very close to the theoretical size of the pattern (XYZ), 8000 µm × 8000 µm × 210 µm. The scaffold fabricated with 40% infill density parameter presented 225 homogenous microenvironments (void spaces or pores) with an actual size (XYZ) of 251.6 ± 11.2 µm × 245.9 ± 16.9 µm × 210 ± 9 µm, compared to the theoretical size of 250 µm × 240 µm × 210 µm. The overall porosity represented approximately 29.7% of the scaffold dimension (Figure 5A,B). In addition, the fibers presented a uniform size with a 279.15 ± 19.4 µm layer width. Thus, our findings indicate that the FDM technique reproduced the 3D scaffold pattern with high precision. FDM is one of the most commonly used rapid prototyping techniques in 3D printing; it is used widely in the automotive and aerospace industries, as it is capable of producing models for visualization and design verification, and it is also used in biomedicine, particularly for the fabrication of 3D scaffolds [9,42]. This reliable technology also enables the fabrication of porous scaffolds with complex geometry and controllable porosity [30]. The FDM technique is cost-effective and can be customized to yield scaffolds suitable for specific tissues and organs [43]. In addition, the FDM technique does not require any solvent to reproduce layer-by-layer 3D scaffold designs via computer models from polymeric thermoplastic, ceramic, and metallic materials [44].

EDS data was analyzed to examine the elemental composition of the particles found on the surface of IA3D fibers and their surroundings (Figure 5C). The results clearly confirmed the presence of metallic Au. In this study, we treated GIA3D (14.54 nm, Figure 4C) synthesized from a HAuCl_4_ solution containing 0.25 mM of Au^3+^ ions. It is important to highlight the fact that Au-NPs can be incorporated into scaffold structures to upgrade the biocompatibility of the resultant nanocomposite. This was confirmed recently by proving that Au-NPs-incorporated macroporous scaffolds increased the matrix conductivity of the scaffold and promoted cell adhesion, growth, differentiation, maturation, and morphogenesis [15,16,17].

### 3.5. Morphology of Cells in G2D and GIA3D

**Figure 6:**
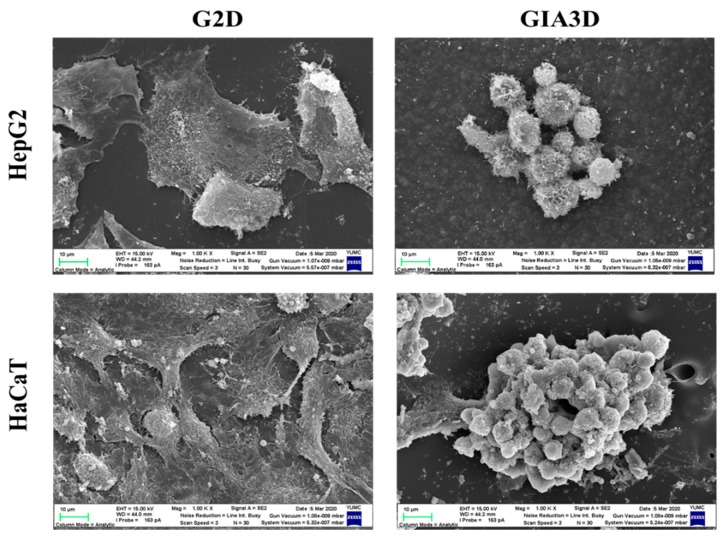
Morphology of human hepatoma cells (HepG2) and HaCaT cells in gold nanoparticles-coated 2D plate (G2D) and Au-NPs-coated industrial ABS scaffold (GIA3D) using SEM. Scale bar: 10 µm.

**Figure 7:**
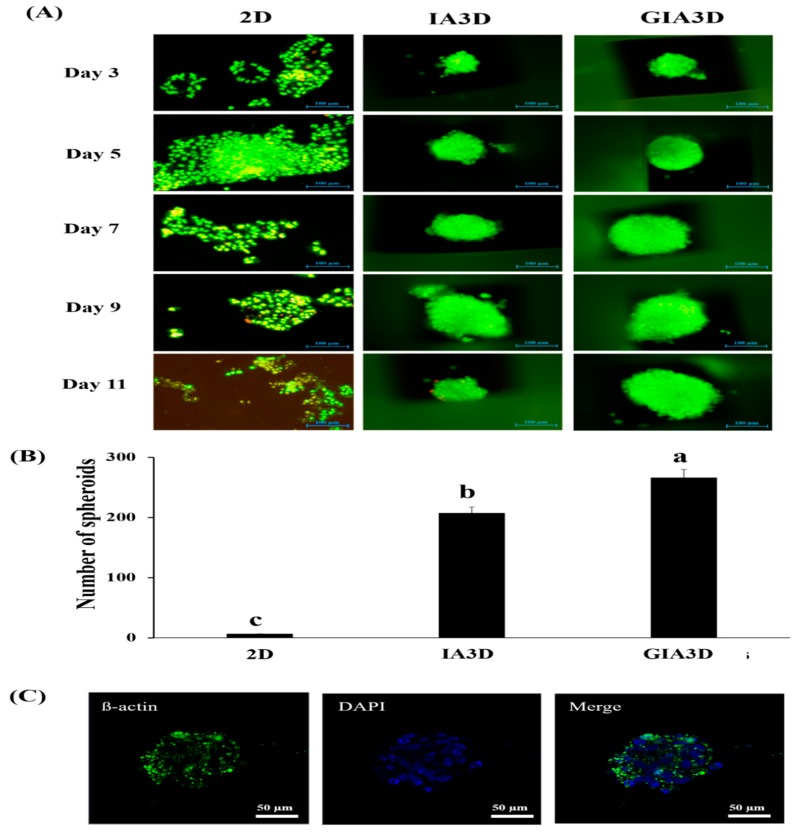
Evaluation of cell proliferation and spheroid stimulation in gold nanoparticle (Au-NPs)-coated industrial acrylonitrile butadiene styrene (ABS) scaffold. (**A**) Live/dead staining micrograph of HepG2 cells cultured for 11 days in two-dimensional culture, industrial ABS scaffold (IA3D), and Au-NPs-coated industrial ABS scaffold (GIA3D); live cells are stained in green. Scale bar, 100 µm. (**B**) Number of HepG2 spheroids formed after a three-day culture in IA3D and GIA3D. Results are expressed as mean ± SD. Significant differences (*p* < 0.05) are represented using different letters (a, b, c). (**C**) Confocal microscopy images of HaCaT cells stained with F-actin (green) using a FITC-conjugated antibody (left micrograph). Nuclei (blue) stained with 4′,6′-diamidino-2-phenylindole (DAPI) (middle micrograph). Merged image of F-actin and DAPI staining (right micrograph). The images were obtained using a Carl Zeiss LSM 800 confocal laser scanning microscope, LSM imaging software (Carl Zeiss). Scale bar 50 µm.

SEM imaging of Figure 6 showed the difference in the morphology of cells grown on GIA3D and G2D. In 2D-plated culture, Au-NPs coating is an efficient technique to reinforce cell adhesion and growth or bind functional compounds for enhancing extracellular matrix properties [45]. The SEM image of HepG2 cells and HaCaT cells cultured on G2D appeared to show a flat and well-spread morphology with microvilli on the surface. In addition, our results showed the similar pattern between G2D and 2D (Figure 6 and Figure 7A). However, a morphologic image of HepG2 and HaCaT cells cultured on GIA3D showed spheroidal structures after 48 h incubation. In addition, GIA3D revealed a significantly greater number of spheroids than that observed for cells grown on G2D. Therefore, our structures allowed for a faster and easier method for culturing HepG2 and HaCaT cells in a spheroid manner.

### 3.6. Enhancement of Long-Term Cell Culture in Surface-Modified GIA3D

To evaluate whether the GIA3D improved the proliferation of cell spheroids, we performed live/dead staining and immunostaining assays (Figure 7). As shown in Figure 7A, the proliferation of tumor HepG2 cells was compared via a long-term culture system (2D versus 3D). Results showed that in the 2D system, the number of living cells markedly increase from 3 days to 5 days of culture but deplete thereafter until 11 days of culture. However, in the 3D culture approach, the number of living cells decreased in the IA3D from 9 days on, and almost all of the cells cultured in GIA3D were viable at 11 days. The substantial increase in cell proliferation that occurred in the 2D culture model raised the number of live cells, resulting in approximately 95% confluence at 5 days, which likely triggered overcrowding-induced cell death.

In addition, in Figure 7B, the number of HepG2 spheroids obtained after 72 h of culture was higher in GIA3D in comparison to IA3D (1.3-fold) and 2D models (38-fold) *p* < 0.05.

To further investigate the cell morphology of normal cell (keratinocytes, HaCaT cells) cultured in GIA3D, we analyzed confocal z-stack sections of individual colonies. After 3 days of culture, HaCaT cells stained with F-actin and blue fluorescence (i.e., DAPI, or 4′,6′-diamidino-2-phenylindole staining) was observed in the nuclei of cells (Figure 7C). Three-dimensional (3D) cell aggregates were found between the pores of the scaffold (Figure 7C). The spheroidal structure of HaCaT cells appeared rapidly in GIA3D after 3 days of incubation.

In recent reports, the incorporation of Au-NPs in scaffold structure promotes cell growth and viability [15]. Au-NPs are known to positively influence the proliferation, maturation, and differentiation of cells in 3D scaffold culture. For instance, the growth and proliferation of neurons and cardiac cells were boosted when cells were cultivated on Au-NPs-decorated electrospun nanofiber scaffolds and within coiled fiber scaffolds embedded with gold nanoparticles, respectively [16,18,19]. Additionally, the Au-NPs-induced activation of cell adhesion, growth, and proliferation is strongly correlated with the particles’ shape and size. Zeng et al. showed that the spherical nano-size of Au-NPs (15-nm), ranging from 0 to 200 pM, did not negatively influence HepG2 cell growth even at their highest tested dose, whereas a larger particle size (30 nm) decreased the viability of cells [46]. Furthermore, Huang et al. proved that 15 nm Au-NPs are not able to penetrate deeply into tumor spheroids [47]. Herein, we have coated the surface of IA3D with a similar spherical particle size (14.54 nm) of Au-NPs (Figure 4C). Interestingly, instead of inducing a cytotoxic effect or decreasing the proliferation of tumor HepG2 cells, Au-NPs promoted cell growth and proliferation. Additionally, other studies have also demonstrated an increase in the number of spheroids found in cells cultured with Au-NPs, including Pavlovich et al. 2016, which reported that small concentrations of nanoparticles (1–3 μg/mL) with 15 nm diameters stimulated multicellular spheroid formation by HT29 (colorectal carcinoma cell line) and SPEV Cells (embryonic porcine kidney epithelial inoculated cell line) [48].

Our findings suggest that the chosen size of Au-NPs, as well as the coating methods, are effective in promoting the proliferation of the cancer model HepG2 cells, in addition to accelerating the formation and growth of HaCaT spheroids. A similar pattern of results was obtained by Chandrasekaran et al. by culturing the HaCaT cells in microbubbles under standard conditions. They observed a compact 3D spheroidal morphology of HaCaT cells within 2–3 days [49]. Our findings may suggest that Au-NPs are useful tools for improving the biocompatibility of industrial materials such as IA3D and serve to broaden the application of this kind of material in the biomedical field. Herein, we report that our newly engineered GIA3D facilitate cell proliferation and the formation of 3D cell spheroids.

### 3.7. Improvement of Cell Viability in Surface-Modified GIA3D

**Figure 8:**
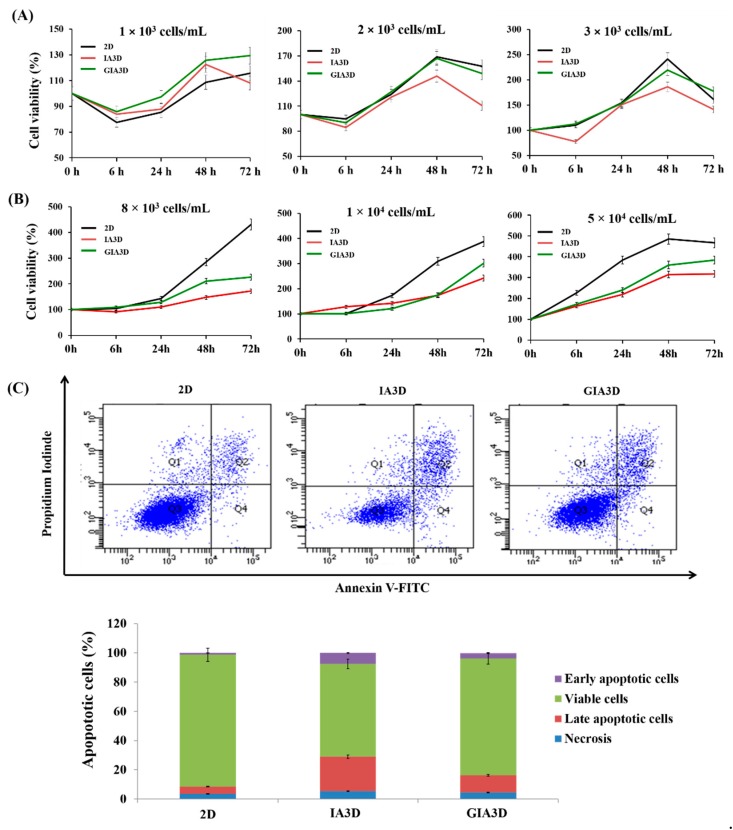
Evaluation of HepG2 and HaCaT cells viabilities. (**A**,**B**) Viabilities of HepG2 cells, HaCaT cells in two-dimensional culture, industrial acrylonitrile butadiene styrene (IA3D), and gold nanoparticle-coated ABS scaffold (GIA3D). Cells were seeded at a density ranging from 1 × 10^3^–3 × 10^3^ cell/mL for HepG2 cells to 8 × 10^3^–5 × 10^4^ cell/mL. Viabilities were measured at 0 h, 6 h, 24 h, 48 h, and 72 h of culture. The percentage (%) of viable cells was calculated and plotted. Results are expressed as mean ± SD, n = 3. (**C**) Determination of the apoptotic HaCaT cell populations by Annexin V-FITC/propidium iodide staining in 2D culture, IA3D, and GIA3D. Cells were cultured at a density of 5 × 10^4^ cells/mL for 3 days. The experiments were performed in triplicate. After 3 days of culture, IA3D induced cell apoptosis (31.2%), which was lower in the GIA3D scaffold (15.3%) and minimal in 2D culture (9.2%).

To confirm the improved biocompatibility of GIA3D, we assessed the viability of cancer HepG2 cells and normal HaCaT cells by culturing the cells in a 2D system, IA3D, and GIA3D for 6, 24, 48, and 72 h (Figure 8). For HepG2 cells, the three cell culture types exhibited different patterns of cell growth according to the cell seeding density and incubation time (Figure 8A). Cell viability curves showed a slight increase in the quantity of cells from 0 h (100%) to 6 h only for cells seeded at 3 × 10^3^ cell/mL in GIA3D (113%) and 2D culture (110%). By contrast, for cells seeded at 1 × 10^3^ cell/mL and 2 × 10^3^ cell/mL, cell viability decreased regardless of cell culture type at 6 h of culture. Afterwards, from 6 to 48 h, cell viability notably increased in all cell culture types according to cell density. For cells seeded at a density of 3 × 10^3^ cell/mL, cell viabilities were 219% in GIA3D versus 241% in 2D and 186% in IA3D at 48 h. Intriguingly, at 72 h, the viabilities of HepG2 cells significantly declined for seeding densities of 2 × 10^3^ cell/mL and 3 × 10^3^ cell/mL regardless of the cell culture type, except in the GIA3D culture at 3 × 10^3^ cell/mL and 1 × 10^3^ cell/mL.

The growth of HaCaT cells in the three cell culture types increased according to the cell density and incubation time (Figure 8B). There was a slight increase in cell viability between 0 and 24 h of culture for cells seeded at 8 × 10^3^ cell/mL and 1 × 10^4^ cell/mL regardless of cell culture type, whereas for densities of 5 × 10^4^ cell/mL, cell viabilities notably increased only after 6 h of culture (172% in GIA3D versus 226% in 2D and 162% in IA3D). Moreover, from 24 to 72 h of culture, the viability of HaCaT cells was higher in 2D culture regardless of cell density, followed by the GIA3D culture. For cells seeded at 5 × 10^4^ cell/mL, cell viabilities after 72 h of culture were 384% in GIA3D versus 431% in 2D and 317% in IA3D cultures. Thus, we used cell densities of 3 × 10^3^ cell/mL and 5 × 10^4^ cell/mL for cancer HepG2 cells and normal HaCaT cells in drug screening experiments, respectively.

Apoptotic cells were quantified by Annexin V-FITC staining in HaCaT cells. As shown in Figure 8C, 31.2% of apoptotic cells were observed in the IA3D as opposed to the GIA3D (15.3%) and 2D culture groups (9.2%). These results also confirmed that Au-NPs increase the biocompatibility of the IA3D, improving the rate of cell proliferation and protecting HaCaT cells from apoptosis. Our knowledge of the apoptotic process is limited without assays to better understand the mechanism of apoptotic induction. Breslin et al. found that apoptotic markers including caspase 3, caspase 7, and caspase 9 are increased in 3D cells compared to 2D cells [50]. Similarly, our findings demonstrated an increase of apoptotic cells in 3D cultured when compared with the 2D culture.

Together, the present findings confirm that the cytotoxicity induced in the IA3D was reduced with Au-NPs coating; thus, Au-NPs improved the biocompatibility of IA3D. Additionally, we found that the GIA3D was more efficient than the 2D culture system in maintaining the viability of HepG2 cancer cells, while in normal HaCaT cell cultures, higher viability was observed in 2D culture as opposed to GIA3D. These findings support the notion that the type of material and cell-type are critical to preventing adverse effects in 3D cell culture [37]. In 2D culture, cells grow continuously when there is enough space, but the 3D culture systems do not follow the same growth pattern [51]. Due to the tight structure of cell spheroids in the 3D culture, the amount of oxygen and nutrients received by cells in the center of the structure is reduced in comparison to the outer cells, which can lead to the death and necrosis of cells in the core of structure. However, the outer cell layers of the spheroid undergo a higher proliferation rate [51]. This may explain why the cell viability in 3D cultures is likely to be reduced compared to 2D cultures. Rabionet et al. demonstrated that breast cancer cells cultured in 15% PCL and 7.5% PCL scaffolds were less proliferative in comparison to cells cultured in a monolayer [52]. In addition, the proliferation of a range of colon cancer cell lines (Caco-2, DLD-1, HT- 29, SW-480, LoVo, COLO-205, and COLO-206f) grown in 3D using Matrigel was decreased compared to the growth observed in 2D cells [53].

### 3.8. Cytotoxicity of Drugs in HepG2 and HaCaT Cells Cultured on GIA3D

**Figure 9:**
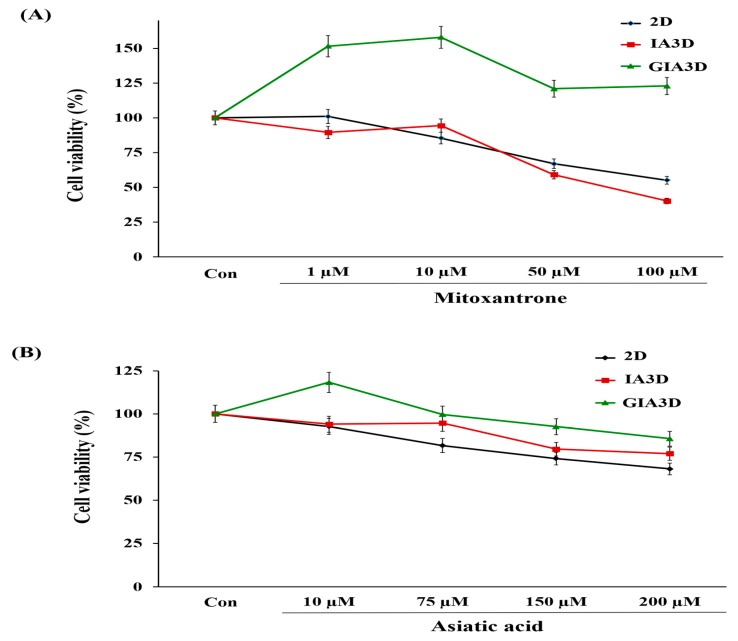
Cytotoxic effects of drugs on HepG2 cells. (**A**,**B**) Effects of mitoxantrone and asiatic acid drugs on HepG2 cells cultured in two-dimensional culture, industrial acrylonitrile butadiene styrene, and gold nanoparticle-coated ABS scaffold. The cells were seeded at 3 × 10^3^ cell/mL, incubated for 72 h afterward, and treated with mitoxantrone (1–100 µM) or asiatic acid (10–200 µM) for 24 h. The relative *cell viabilities were calculated* as the percentage of untreated cells. Results are expressed as mean ± SD, n = 3.

The 3D cell models have become useful tools for mimicking in vivo conditions, and they can be used for more effective drug screening applications [1]. To evaluate the sensitivity of both cancer and normal cells to drugs, we treated the cultured cancer HepG2 cells and normal skin HaCaT cells with mitoxantrone and asiatic acid (AA) in IA3D and GIA3D as well as 2D culture. In cancer cells, mitoxantrone treatment (1–100 µM) resulted in a dose-dependent reduction in the viability of HepG2 cells cultured in 2D culture and IA3D in comparison with untreated cells (Figure 9A).

However, in the GIA3D, the percentage of viable cells remained higher than that observed in the untreated cells regardless of the drug dose (Figure 9A). In fact, mitoxantrone linearly decreased the viability of cells up to 55.2% in 2D culture and 40.2% in IA3D culture at a dose of 100 µM, while in the GIA3D, cell viability was increased (*p* < 0.05). The AA (0–200 µM) screening test in HepG2 cells demonstrated that 10 µM of the drug markedly increased the viability of cells only when cultured in GIA3D (118%) in comparison with untreated cells. However, no significant difference was observed in all three cell culture types when cells were intoxicated with 10 µM and 75 µM of AA (*p* < 0.05) (Figure 9B). By contrast, a notable decrease in cell viability was noticed in cells treated with 150 and 200 µM of AA in 2D culture (74.1% and 68.1%, respectively) and IA3D (79.5% and 76.9%, respectively) (*p* < 0.05). Interestingly, in cells cultured in the GIA3D, no significant changes were observed with the same concentrations of 150 and 200 µM (*p* < 0.05). Our findings show that cancer HepG2 cells and normal HaCaT cells were less sensitive to mitoxantrone and AA in GIA3D culture than in IA3D and 2D cultures.

**Figure 10:**
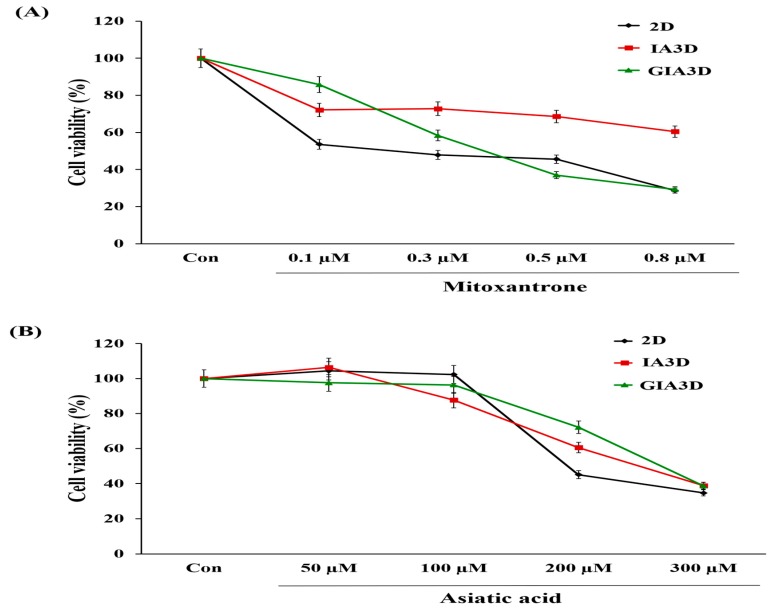
Cytotoxic effects of drugs on HaCaT cells. (**A**,**B**) Effects of mitoxantrone and asiatic acid drugs on HaCaT cells cultured in two-dimensional culture, industrial acrylonitrile butadiene styrene, and gold nanoparticle-coated ABS scaffold. The cells were seeded at 5 × 10^4^ cell/mL, incubated for 72 h afterward, treated with mitoxantrone (0.1–0.8 µM), or asiatic acid (50–300 µM) for 24 h. The relative *cell viabilities were calculated* as the percentage of untreated cells. Results are expressed as mean ± SD, *n* = 3.

In addition, the sensitivity to mitoxantrone and AA was investigated in HaCaT cells cultured in IA3D, GIA3D, and 2D cultures (Figure 10A,B). The viability of HaCaT cells treated with 0.1 µM mitoxantrone dropped significantly to 85.76% in GIA3D, 53.59% in 2D, and 72.15% in IA3D culture when compared with corresponding untreated cells. However, with mitoxantrone doses of 0.3 µM and 0.8 µM, cell viabilities were higher in IA3D culture, followed by GIA3D and 2D culture (60.5% in IA3D versus approximately 29% in GIA3D and 2D for 0.8 µM of AA). On the other hand, in AA intoxication, no significant changes were observed in the viabilities of HaCaT cells treated with 50 and 100 µM in all cell culture types compared with untreated cells. However, 200 and 300 µM of AA notably decreased cell viability in all cell culture types up to approximately 34–38%.

Mitoxantrone is a quinone-based drug recognized as a FDA-approved compound for use as an anticancer agent [54]. For example, the drug was shown to exhibit cytotoxic activities in liver carcinomas [55]. For instance, in hepatocellular carcinoma HepG2 cells, mitoxantrone have been shown to induce cell death with autophagy involvement [55]. In the current study, mitoxantrone reduced the viability of HepG2 cells cultured in 2D and IA3D, whereas no toxicity was observed in cells cultured in GIA3D, even at the highest tested concentration (100 µM). AA is a pentacyclic triterpene that is mainly found in *Centella asiatica*, which is proven to induce a significant anti-proliferative effect and death in cancer cells such as HepG2 [56,57]. Our findings show that the AA treatment resulted in a greater reduction in cell viability of HepG2 cells in 2D culture than that observed in both IA3D. Especially in the GIA3D, cells were less sensitive to AA compared with IA3D. Hickman et al. revealed that the tight structure of HepG2 cell aggregates in 3D scaffold structures limits the accessibility of the drug to some cells [58]. By contrast, in 2D culture, the weak interactions between cells [59] (which appear in a diffuse formation) may facilitate the distribution of the drug to cells. Moreover, drug sensitivity variation among 2D and 3D cultures was shown to be cell-type dependent. Our findings revealed that the GIA3D is a potent candidate for cancer drug screening. Furthermore, the 3D culture systems mimic the in vivo environment better than traditional 2D cell culture due to the ability of the cells to form cell–cell interactions and develop into 3D structures in comparison with cell culture in a flat Petri dish [60]. Nevertheless, further experiments are needed to better understand the mechanisms involved in the cytotoxicity of drugs in our 3D scaffolds.

## 4. Conclusions

To our knowledge, we report for the first time that IA3D can be safely used for stimulating cell proliferation, spheroid formation, and drug screening applications. The experimental conditions were optimized to accelerate the spheroid formation time for the cancer cells HepG2 (in 2 days) and keratinocytes HaCaT cells (in 3 days). Interestingly, GIA3D increased the number of HepG2 spheroids by 1.3-fold and reduced the number of apoptotic HaCaT cells (15.3% in GIA3D versus 31.2% in IA3D) when compared with IA3D. Additionally, the GIA3D improved cell proliferation and highly reduced the sensitivity of HepG2 cells to mitoxantrone and asiatic acid drugs when compared with IA3D and 2D culture. These results indicated that the proposed 3D structures could potentially be used as a diagnostic system, anticancer evaluation, and cosmetic testing application or can be utilized to prolong various cell functions in culture. Thus, our results suggest that this cost-effective and attractive 3D scaffold may be used in vitro for cellular biology studies and drug screening applications to mimic in vivo phenomena.
